# The influence of sample geometry and size on porcine aortic material properties from uniaxial tensile tests using custom-designed tissue cutters, clamps and molds

**DOI:** 10.1371/journal.pone.0244390

**Published:** 2021-02-08

**Authors:** Ming Pei, Donghua Zou, Yong Gao, Jianhua Zhang, Ping Huang, Jiawen Wang, Jiang Huang, Zhengdong Li, Yijiu Chen

**Affiliations:** 1 West China School of Basic Medical Sciences & Forensic Medicine, Sichuan University, Chengdu, Sichuan Province, China; 2 Shanghai Key laboratory of Forensic Medicine, Academy of Forensic Science, Ministry of Justice, Shanghai, China; 3 Institute of Forensic Science, Xuzhou Public Security Bureau, Xuzhou, Jiangsu Province, China; 4 School of Forensic Medicine, Guizhou Medical University, Guiyang, Guizhou Province, China; 5 School of Mechanical Engineering, University of Shanghai for Science and Technology, Shanghai, China; 6 Department of Forensic Medicine, School of Basic Medical Sciences, Fudan University, Shanghai, China; China University of Mining and Technology, CHINA

## Abstract

The aim of this study was to identify the influence of specimen geometry and size on the results of aortic uniaxial tensile tests using custom-designed tissue cutters, clamps and molds. Six descending thoracic aortas from pigs were used for rectangular sample tests, in which the circumferential and axial specimens had widths of 6 mm, 8 mm and 10 mm. The other six aortas were used for the dog-bone-shaped sample tests and were punched into circumferential, axial and oblique specimens with widths of 2 mm, 4 mm and 6 mm. We performed uniaxial tensile tests on the specimens and compared the test results. The results showed that mid-sample failure occurred in 85.2% of the dog-bone-shaped specimens and in 11.1% of the rectangular samples, which could be caused by Saint-Venant’s principle. Therefore, rectangular specimens were not suitable for aortic uniaxial tensile testing performed until rupture. The results also showed that the size effect of the aorta conformed to Weibull theory, and dog-bone-shaped specimens with a width of 4 mm were the optimal choice for aortic uniaxial tensile testing performed until rupture.

## Introduction

Aortic rupture is an important cause of death in forensic practices with a mortality rate as high as 80%-94.4% [1–3]. This phenomenon is often caused by internal diseases (such as aortic dissection [4, 5], aneurysms [6–8], and atherosclerosis [9]) or blunt force [10–12] and can occur during cardiopulmonary resuscitation [13]. It is difficult to identify the cause of rupture or the degree of participation of trauma when trauma and disease coexist. The biomechanical analysis of injury based on the finite element method is important when studying the mechanism of aortic injuries, and one of the key research bases is the material property data of the aorta.

Uniaxial tensile tests are mechanical property tests that are widely used to study the mechanical properties of aortic tissues [14–32]. However, the unique methodological procedures used in mechanical testing of human aortic tissue are not well defined or widely accepted, especially in regard to the geometry and size of the specimens. Most specimens in previous studies were processed into rectangles [14–27], whereas some were processed into dog-bone shapes [28, 29, 31, 32]. Guinea GV [33] and García-Herrera CM et al. [30] processed their specimens into dog-bone shapes but clamped them to the beginning of a rectangle, which was equivalent to tensile tests of the rectangular samples.

Consequently, the reported experimental results are often incomparable, especially in regard to the failure stress and strain. Table 1 summarizes some of the previous studies documented in the literature regarding the failure stress and strain of intact artery walls. This table lists the source, geometry and size of the samples and provides the failure stress and strain values of the samples under uniaxial tensile load. This table is not a complete summary of all uniaxial rupture tests of intact artery wall samples published in the literature; however, it is intended to provide a representative overview that illustrates that failure stress and strain values are discrete.

**Table 1:** Overview of uniaxial tensile test results on aorta full-thickness tests performed until failure.

Author	Tissue description	Shape	Dimensions	Direction	Sample (n)	Failure stress (kPa)	Failure strain (-)
Mohan & Melvin, 1982 [28]	DTA	Dog-bone	19.05 mm×6.35 mm or 7.87 mm×4.57 mm*	Circ	18	1720±890	Stretch: 1.53±0.28
				Long	18	1470±910	Stretch: 1.47±0.23
Sherebrin, 1989 [14]	Upper thoracic aorta	Rectangle	30 mm×5 mm	Circ	9	177±104	Strain: 0.20~0.66
				Long	9	184±90	Strain: 0.18~0.49
Raghavan et al., 1996 [16]	ABA	Rectangle	40 mm×10 mm	Long	7	2014±394	-
	ABA AN			Circ	16	1019±160	-
				Long	45	864±120	-
Vorp et al., 2003 [17]	ASA	Rectangle	30 mm×8 mm	Circ	7	1800±240	-
				Long	7	1710±140	-
	ASA AN			Circ	23	1180±120	-
				Long	17	1210±90	-
Vallabhaneni et al., 2004 [18]	Aortic aneurysms	Rectangle	30–40 mm×4 mm	Long	96	530±20	Strain: 0.3±0.02
	Aortic			Circ	52	610±70	Strain: 0.29±0.04
				Long		1300±110	Strain: 0.33±0.04
Di Martino et al., 2006 [19]	ABA AN: unruptured	Rectangle	25 mm×7 mm	Circ	26	820±90	-
	ABA AN: ruptured				13	540±60	-
Raghavan et al., 2011 [23]	ABA AN: unruptured	Rectangle	Width:4 mm	Circ	2	650±90	Engineering strain: 0.38±0.07
				Long	7	980±230	Engineering strain: 0.36±0.09
	ABA AN: ruptured			Long	4	950±280	Engineering strain: 0.39±0.09
García-Herrera et al., 2012 [30]	ASA <35years	Dog-bone	10 mm×2 mm*	Circ	9	2180±240	Stretch: 2.35±0.1
				Long	9	1140±100	Stretch: 2.0±0.1
	ASA >35years			Circ	12	1200±200	
				Long	12	660±70	
	ASA BAV			Circ	11	1230±150	Stretch: 1.80±0.08
				Long	11	840±100	Stretch: 1.58±0.06
	ASA AN TAV			Circ	11	1190 ±130	
				Long	11	880±120	
Reeps et al., 2013 [25]	ABA AN	Rectangle	20 mm×8 mm	-	50	Engineering stress: 1063±190	-
Pichamuthu et al., 2013 [26]	ASA AN BAV	Rectangle	-	Circ	23	1656±98	Engineering strain: 0.92±0.04
				Long	23	698±31	Engineering strain: 0.63±0.02
	ASA AN TAV			Circ	15	961±61	Engineering strain: 0.61±0.04
				Long	15	540±37	Engineering strain: 0.47±0.03
Ferrara et al., 2016 [31]	ASA AN	Dog-bone	-	Anterior Circ	37	1440±700	Strain: 0.29±0.12
				Anterior Long	34	940±490	Strain:0.29±0.11
				Posterior Circ	31	1850±700	Strain:0.30±0.09
				Posterior Long	19	740±180	Strain:0.27±0.07

Note: ABA = abdominal aorta; AN = aneurysmatic, ASA = ascending aorta, BAV = bicuspid aortic valve, Circ = circumferential, DTA = descending thoracic aorta, Long = longitudinal, TAV = tricuspid aortic valve, and

* = narrowed region.

Mohan and Melvin [28] performed pioneering research and measured circumferential and axial failure stresses and strains under quasi-static and dynamic conditions using dog-bone-shaped samples that were 6.35 or 4.57 mm in width. The circumferential and longitudinal tensile strength values under quasi-static conditions were 1.7±0.2 and 1.5±0.2 MPa, respectively. In studies using healthy thoracic aortas, Sherebrin et al. [14] reported lower values, of which the failure stresses in the circumferential and longitudinal directions were 177±104 kPa and 184±90 KPa, respectively, by using rectangular samples of 5 mm width. However, García-Herrera et al. [30] reported higher values, of which the values in circumferential and longitudinal directions were 2180±240 kPa and 1140±100 kPa, respectively.

In other studies [16, 26, 28, 30, 31], the circumferential failure stress was greater than the axial stress in both normal aortic walls and aortic aneurysm walls. However, a few studies [18, 23] reported the opposite results, whereas Vorp et al. [17] found no significant difference in the different directions. The ultimate stress of the aortic aneurysm walls was found to be significantly lower in the two directions than that in the healthy aortic walls [16–18]. Layer-specific characterization of human aortas has also been studied in recent years, and the results vary [34–40].

Therefore, it is still a challenge to find a scientific and reasonable experimental procedure to accurately characterize the mechanical properties of aortic tissues. The objective of this study is to identify the influence of specimen geometry and size and determine the optimal specimen by comparing the test results of specimens of different geometries and sizes processed by custom-designed tissue cutters, clamps and molds.

## Materials and methods

### Specimen collection and initial processing

All aortic tissue specimens were obtained in accordance with the guidelines of the review board of the Academy of Forensic Science. Twelve descending thoracic aortas from five-month-old pigs were harvested from a local slaughterhouse (Shanghai Yunong Meat Products Co., Ltd., Shanghai) and immediately wrapped in saline-soaked gauze, after which they were stored in an ice-filled box for transport. The aortas reached the laboratory within two hours after harvesting. All samples were frozen to -80°C until experimental testing. Among them, 6 aortas were used for rectangular sample tests and the other 6 aortas were used for the dog-bone-shaped sample tests. Before the test, the samples were removed from the refrigerator at -80°C and immersed in normal saline without Ca^2+^ at 4°C overnight. Then, the loose connective tissue attached to the adventitia was carefully removed so that only the aortic wall itself remained for testing after equilibrating at room temperature.

### Mechanical testing using rectangular samples

**Fig 1:**
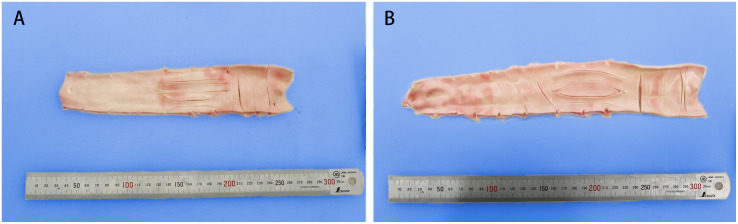
Distribution of specimens: (A) rectangular specimens and (B) dog-bone-shaped specimens.

Six descending thoracic aortas were used for this test. Rectangular specimens that are 6 mm, 8 mm and 10 mm in width (the 6-mm-wide specimens were punched into a dog-bone shape, but they were clamped to the beginning of the narrow zone) were punched from the aortic wall in the circumferential and axial orientation with custom-designed tissue cutters, and the aortic ostia were avoided. Due to the large circumferential diameter at the proximal portion of the descending aortas, three circumferential tissue strips that are 6 mm, 8 mm and 10 mm in width were punched there. Below these strips, three longitudinal specimens were acquired (Fig 1A).

Six specimens were taken from each aorta, and a total of 36 specimens were taken from 6 aortas. The samples with the same width and direction were divided into one group and named RC_6_, RC_8_, RC_10_, RL_6_, RL_8_ and RL_10_. There were 6 specimens for each group. R indicated rectangular, C indicated circumferential, L indicated longitudinal, and the number indicated the width of the test area in mm. For example, RC_6_ represented rectangular circumferential samples with a 6-mm-wide test area. Note that the specimens of each width should appear to be equal at each position in each segment. Table 2 shows a detailed description of the sample groups.

**Table 2:** Description of sample groups.

Proximal	A			Rectangular sample groups	RC_6_	RC_8_	RC_10_	RL_6_	RL_8_	RL_10_	-	-	-
	B												
	C												
Middle	A	B	C	Dog-bone-shaped sample groups	DC_2_	DC_4_	DC_6_	DL_2_	DL_4_	DL_6_	DO_2_	DO_4_	DO_6_
Distal				1	C_A_	C_B_	C_C_	L_A_	L_B_	L_C_	O_A_	O_B_	O_C_
	A			2	C_A_	C_B_	C_C_	L_A_	L_B_	L_C_	O_A_	O_B_	O_C_
	B			3	C_B_	C_C_	C_A_	L_B_	L_C_	L_A_	O_B_	O_C_	O_A_
	C			4	C_B_	C_C_	C_A_	L_B_	L_C_	L_A_	O_B_	O_C_	O_A_
Position diagram of samples on the aortic wall				5	C_C_	C_A_	C_B_	L_C_	L_A_	L_B_	O_C_	O_A_	O_B_
				6	C_C_	C_A_	C_B_	L_C_	L_A_	L_B_	O_C_	O_A_	O_B_

Note: C = circumferential. L = longitudinal. O = oblique. _A_, _B_ and _C_ indicated position of samples on the aortic wall.

**Fig 2:**
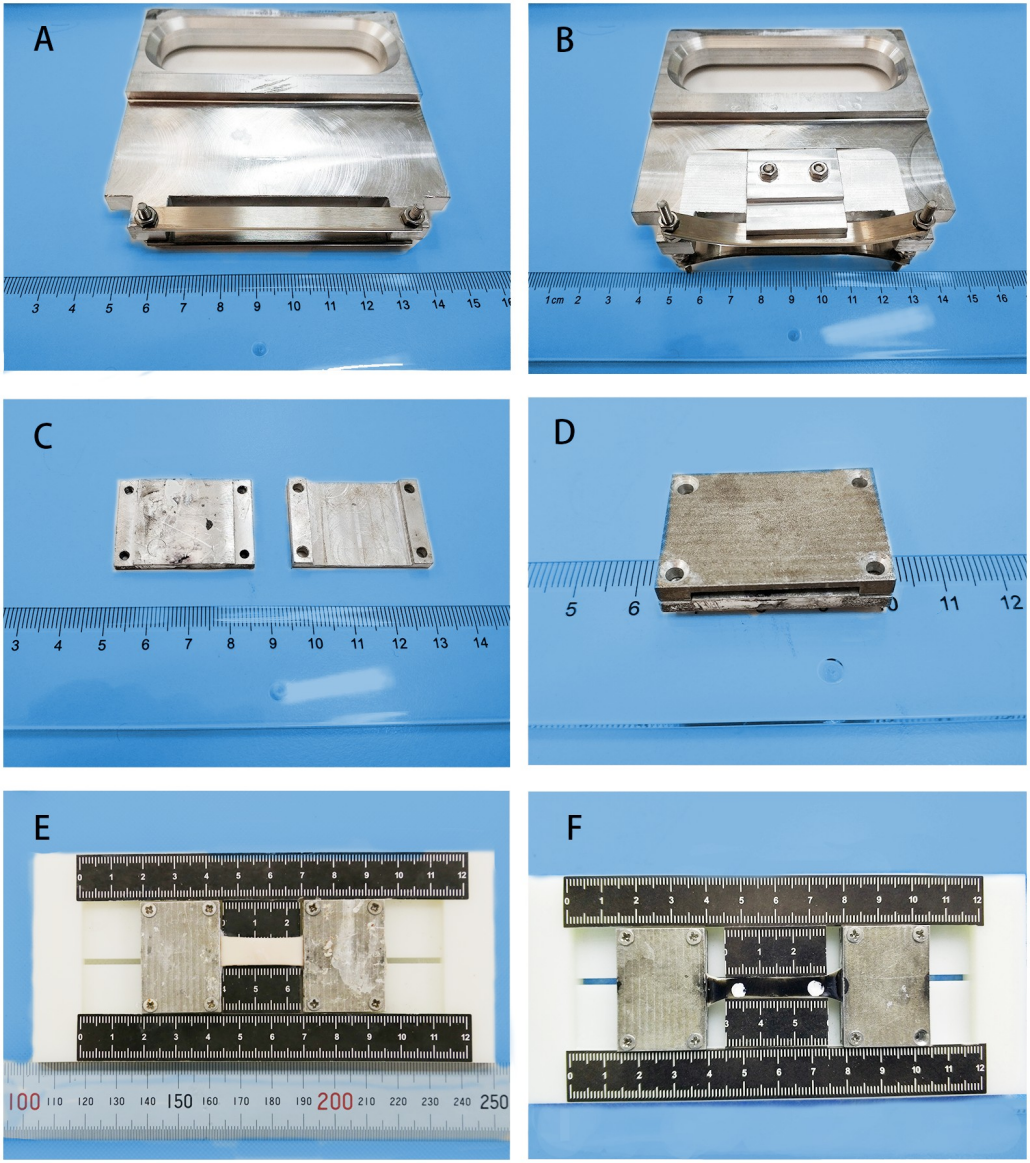
Custom-designed cutters, clamps and molds used for specimen processing. (A) Customized cutter for rectangular sample with a 10 mm width. (B) Customized cutter for dog-bone-shaped samples with a 6 mm width. (C) Open state of the customized clamp. (D) Closed state of the customized clamp. (E) Processed RC_8_, for which the aspect ratio is 3:1. (F) Processed DC_6_, for which the aspect ratio of the test zone is 4:1.

The circumferential samples were limited by the diameter of the aorta, which was approximately 50 mm, ensuring that the aspect ratio of the test area was 3:1 [21, 22]. The lengths of the axial samples were generally not less than 80 mm, ensuring that the aspect ratio of the test area was 4:1. The width and thickness of the samples were photographed vertically in the front and the side and measured at five locations in the test area using ImageJ software to obtain average values. The two ends of each strip were quickly sandwiched between the custom-designed clamps and coupled with cyanoacrylate while ensuring the unity of the aspect ratio on the custom-designed molds with the scale. The custom-designed cutters, clamps and molds used for specimen processing are shown in Fig 2A–2E.

An Instron 5967 machine was used to perform uniaxial tests on the samples at room temperature. The clamps and samples were mounted in the jaws of the pneumatic grips of the testing machine. The distance between the grips was adjusted, starting from a configuration with some slack, and then the sample was slowly extended until the load cell recorded a tensile force of 0.01 N. That was assumed to be the load-free configuration (initial point). The distance between the grips in this initial point was the original length L_0_. The load and the grip displacement were measured by the testing machine, and the grip displacement was taken as a direct measurement of the elongation of the samples because the rest of the elements were much more rigid than the specimens. Each specimen was preconditioned by applying five cycles with a 20%×L_0_/min displacement rate at L_0_×4% stretch to eliminate the hysteresis effect of tissues and obtain repeatable stress-strain curves. Then, tensile testing was carried out at the same speed until tissue failure. The applied force and corresponding displacement of the grips were collected synchronously and continuously at a sampling rate of 100 Hz until specimen failure. The specimens were kept wet by spraying saline solution before and during the experiment.

### Mechanical testing using dog-bone-shaped samples

Six descending thoracic aortas were used for the test. The aortas were divided into three segments: proximal, middle and distal, with lengths of approximately 6 cm, 8 cm and 8 cm, respectively. The circumferential samples were obtained from the proximal section, the longitudinal section from the middle section, and the oblique 45° samples from the distal section. In each of the three segments, customized cutters were used to punch out three dog-bone-shaped samples, for which the dimensions of the narrow middle part were 12 mm×2 mm, 24 mm×4 mm and 30 mm×6 mm; moreover, note that the aortic ostia were avoided (Fig 1B).

Nine specimens were obtained from 1 aorta, and 54 specimens were obtained from 6 aortas. The specimens with the same width and direction were divided into a group and named DC_2_, DC_4_, DC_6_, DL_2_, DL_4_, DL_6_, DO_2_, DO_4_, and DO_6_. There were 6 specimens for each group. Note that D indicated dog-bone-shaped, C indicated circumferential, L indicated longitudinal, O indicated oblique, and number indicated the width of the test area. For example, DC_2_ represented dog-bone-shaped samples with the circumferential direction, and the width of the test area was 2 mm. Note that the specimens of each width should appear to be equal at each position in each segment. The detailed description of the sample groups is shown in Table 2.

The measurements of the original width and thickness of the test area and the clamping of the specimens were consistent with the previous experiments.

Water-proof black ink was applied to the inner side of the samples after the strips were clamped. Two white paint spots, which were spaced 4 times the width apart at the center of the narrow zone of the specimens, were tracked by the video extensometer to identify the original length L_0_ and displacement of the test area (Fig 2F). If the samples were directly marked, it was difficult to recognize by the video extensometer because the color contrast was not obvious.

An Instron 5967 machine was used to perform the uniaxial tests. At the initial point, the distance between the two markers was the original length, L_0_. The applied force and corresponding displacement of the markers were collected synchronously and continuously at a sampling rate of 100 Hz. The other experimental procedures were consistent with the previous one.

### Evaluation of the mechanical parameters

The applied force and the extension were recorded during each experiment. Combined with the width and thickness of the measured initial state of the specimens, engineering strain, engineering stress, real strain and real stress can be converted. The engineering strain (ε_E_) is elongation (ΔL) divided by the initial length (L_0_):

$$\varepsilon_{E}=\frac{\Delta L}{L_{0}}$$

The engineering stress (σ_E_) is the applied load (F) divided by the initial cross-sectional area (A_0_):

$$\sigma_{E}=\frac{F}{A_{0}}$$

The true strain (ε_T_) is the natural log of the current length (L) divided by the initial length (L_0_):

$$\varepsilon_{T}=\text{ln}\left(\frac{L}{L_{0}}\right)$$

The volume of the aortic wall was assumed to remain unchanged during the elongation process [41], so the current cross-sectional area A was obtained by the equation:

$$A=\frac{A_{0}L_{0}}{L}$$

The true stress (σ_T_) is calculated as the applied load (F) divided by the current cross-sectional area (A).

$$\sigma_{T}=\frac{FL}{A_{0}L_{0}}$$

**Fig 3:**
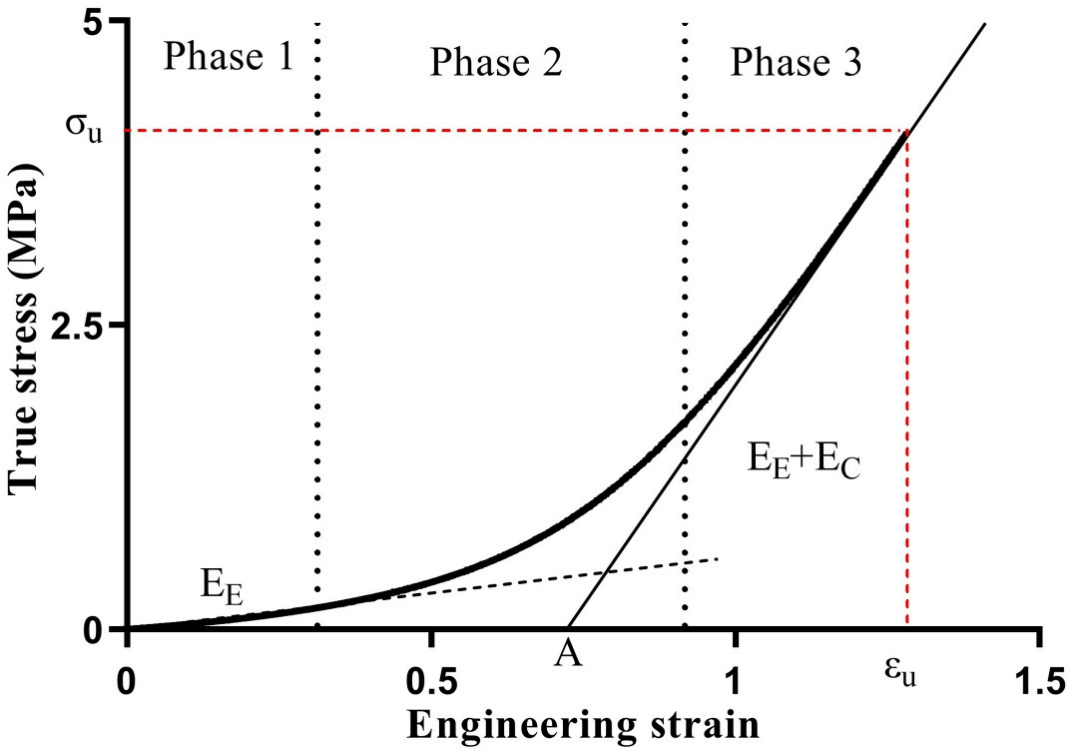
The three presumed phases of the aortic elastic response and the relationship between the elastin fiber modulus, collagen fiber modulus and stress-strain curve.

Because the complex material constitutive model involves more parameter fitting and most of the parameters have no direct mechanical significance, it is not convenient for comparison between groups. To simplify the analysis and facilitate comparisons between the specimens in different groups, the mathematical model introduced by Raghavan et al. [16] was used in this study. The relationship between strain and stress is expressed as follows:

$$\varepsilon =(K+\frac{A}{B+\sigma })\sigma$$

where ε is the engineering strain, σ is the true stress, and K, A, and B are model parameters in the formula. According to the theory, the aorta can be simplified into containing two primary passive load bearing fibers: elastin and collagen. The stress-strain curve is divided into three phases (Fig 3).

In phase 1, the stress is low, and only the elastic fibers are taut (Fig 3, phase 1). As *σ* → 0 and B + σ ≈ B, the formula for the Young’s modulus of elastin (E_E_) can be expressed as follows:

$$E_{E}=\frac{1}{K+\frac{A}{B}}$$

In phase 2, the collagen fibers start contributing to load bearing, and the slope of the stress-strain curve increases gradually (Fig 3, phase 2). Prior to tissue failure, the maximum slope of the final portion of the stress-strain curve corresponds to the combined total stiffness of the elastin and collagen fibers in phase 3 (Fig 3, phase 3). Assume that *B* ≪ *σ*, so that *B* + *σ* ≈ *σ*, and the following formulas can be derived:

$${\text{E}}_{\text{E}}+{\text{E}}_{\text{C}}=\frac{1}{\text{K}}\;\text{and}\;{\text{E}}_{\text{C}}=\frac{\text{A}}{\text{K}(\text{A}+\text{KB})}$$

where E_C_ is the Young’s modulus of collagen. For detail, as hown by Raghavan et al. [16], K is the inverse of (E_E_+E_C_). A is the strain intercept of the final portion (phase 3) of the stress-strain curve (Fig 3), A is inversely proportional to the rate of recruitment of the collagen fibers for a given strain rate, or directly proportional to the average degree of tortuosity of the collagen fibers in the tissue. A can be considered the “recruitment parameter”. The smaller A is, the faster the collagen fibers activate. B is the value of stress at the intersection of the lines defined by the linear responses in phase 1 and phase 3 (dashed and solid lines in Fig 3, respectively). Moreover, ε_u_ and σ_u_ are the ultimate strain and ultimate stress, respectively.

Thus, the stress-strain curve is simplified into three parameters to characterize the main mechanical properties of the aortic wall: K, A and B. Moreover, E_E_ and E_C_ can be deduced from these parameters. Compared with the use of more precise and complex material constitutive relations, this method of selecting parameters for comparison is more meaningful and easier to operate.

### Data processing and statistics

The stress-strain curve was plotted, and the ultimate strain (ε_u_) and ultimate stress (σ_u_) were recorded for each specimen. Using the Levenberg-Marquardt for nonlinear regression (SPSS, Statistics, version 20), the mathematical model $\varepsilon =(K+\frac{A}{B+\sigma })\sigma$ was fit to the experimentally measured stress-strain data obtained for each specimen, yielding the best-fit parameters K, A, and B. E_E_ and E_C_ were deduced from these parameters for each specimen.

According to the previous groups, the values of the parameters are presented as the mean ± standard deviation for each group. The groups were compared by one-way analysis of variance (ANOVA) followed by least significant difference (LSD) multiple comparison tests if the overall comparison was significant. A two-tailed independent Student’s t-test was performed for the circumferential and axial specimens from the groups with a width of 6 mm between the rectangular and dog-bone-shaped groups examined in this study using SPSS v20. Significance was assumed for a p value less than 0.05.

## Results

### Failure point of the specimens

**Fig 4:**
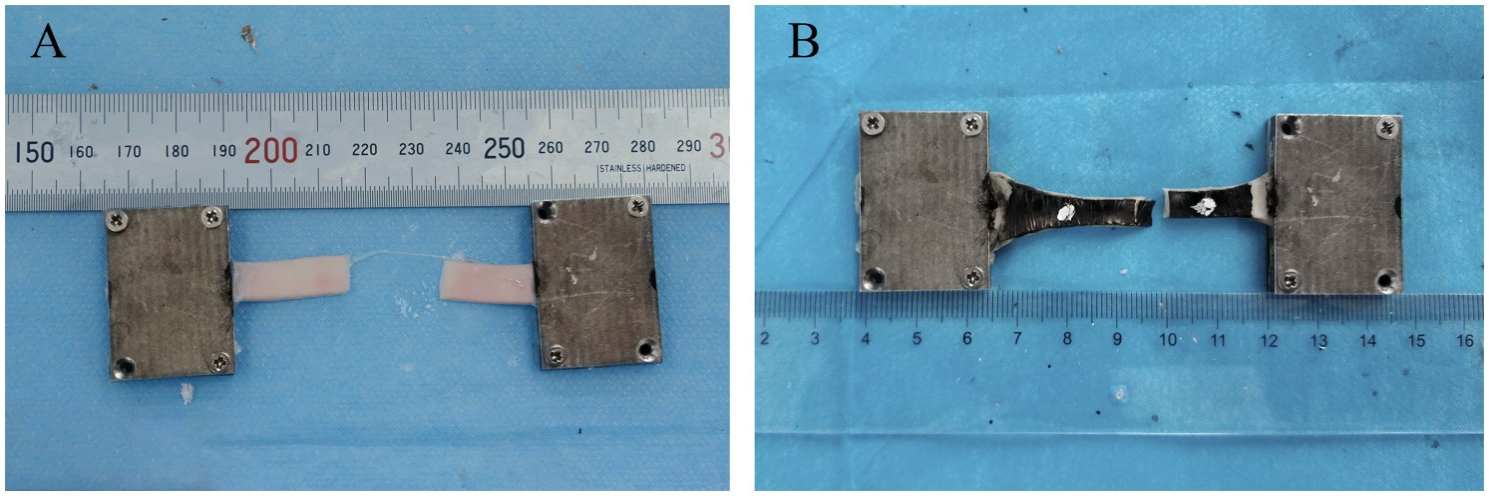
Failed points in the middle of specimens: (A) rectangular specimen and (B) dog-bone-shaped specimen.

Only 11.1% of the rectangular specimens failed in the middle part of the samples (Fig 4A), and 88.9% of them failed in the vicinity of the clamps. In contrast, mid-sample failure occurred in 85.2% of the dog-bone-shaped specimens (Fig 4B), whereas 14.8% of the dog-bone-shaped specimens failed in the vicinity of the clamps.

### Data fitting and comparison

**Fig 5:**
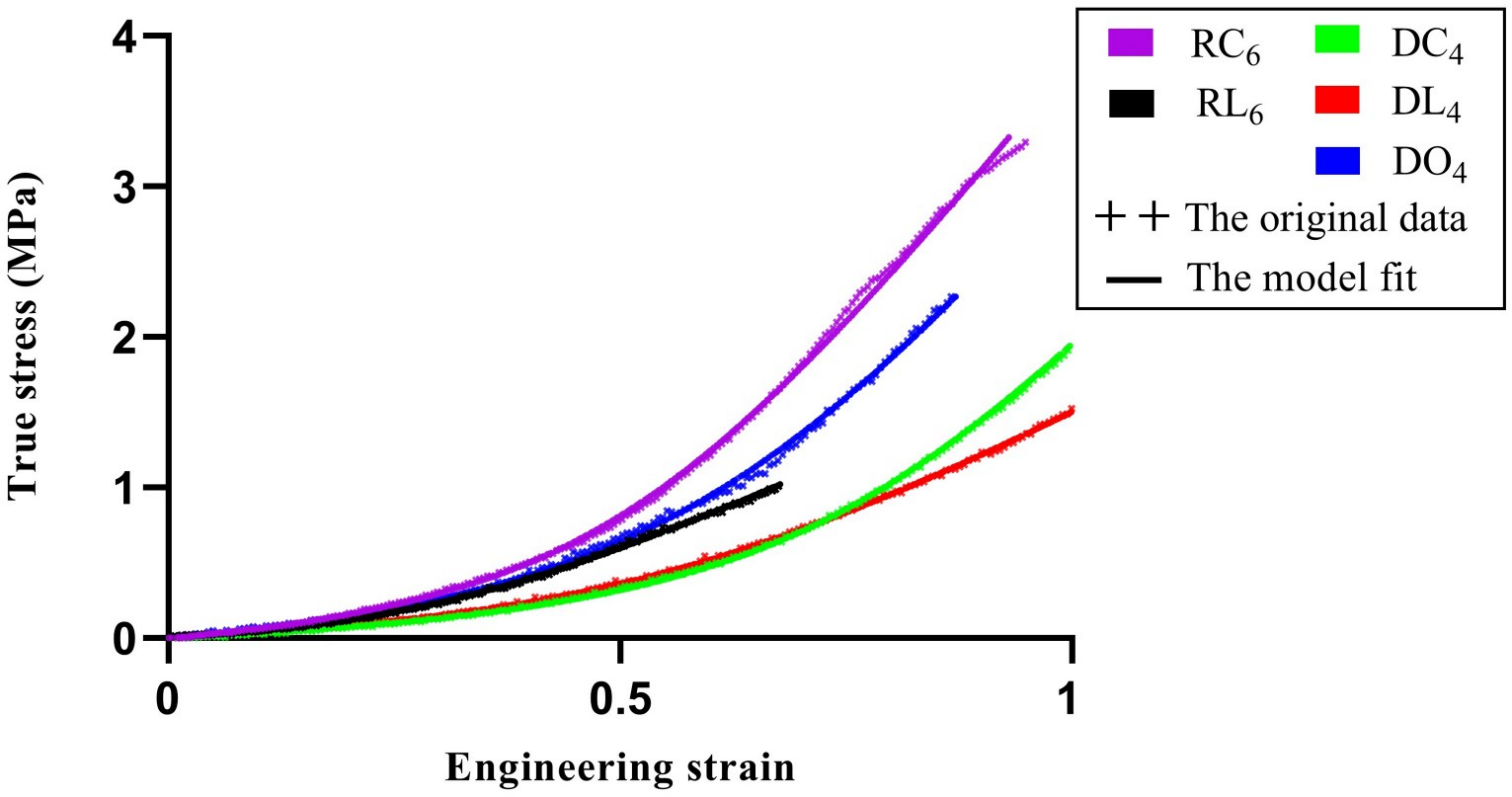
Representative data and model fitting.

The regression of all data sets converged, obtaining the best fitting parameter of each specimen. Fig 5 indicates the representative experimental data of the five groups and the corresponding fit of the model that was selected in the present study using the best fitting parameters.

**Fig 6:**
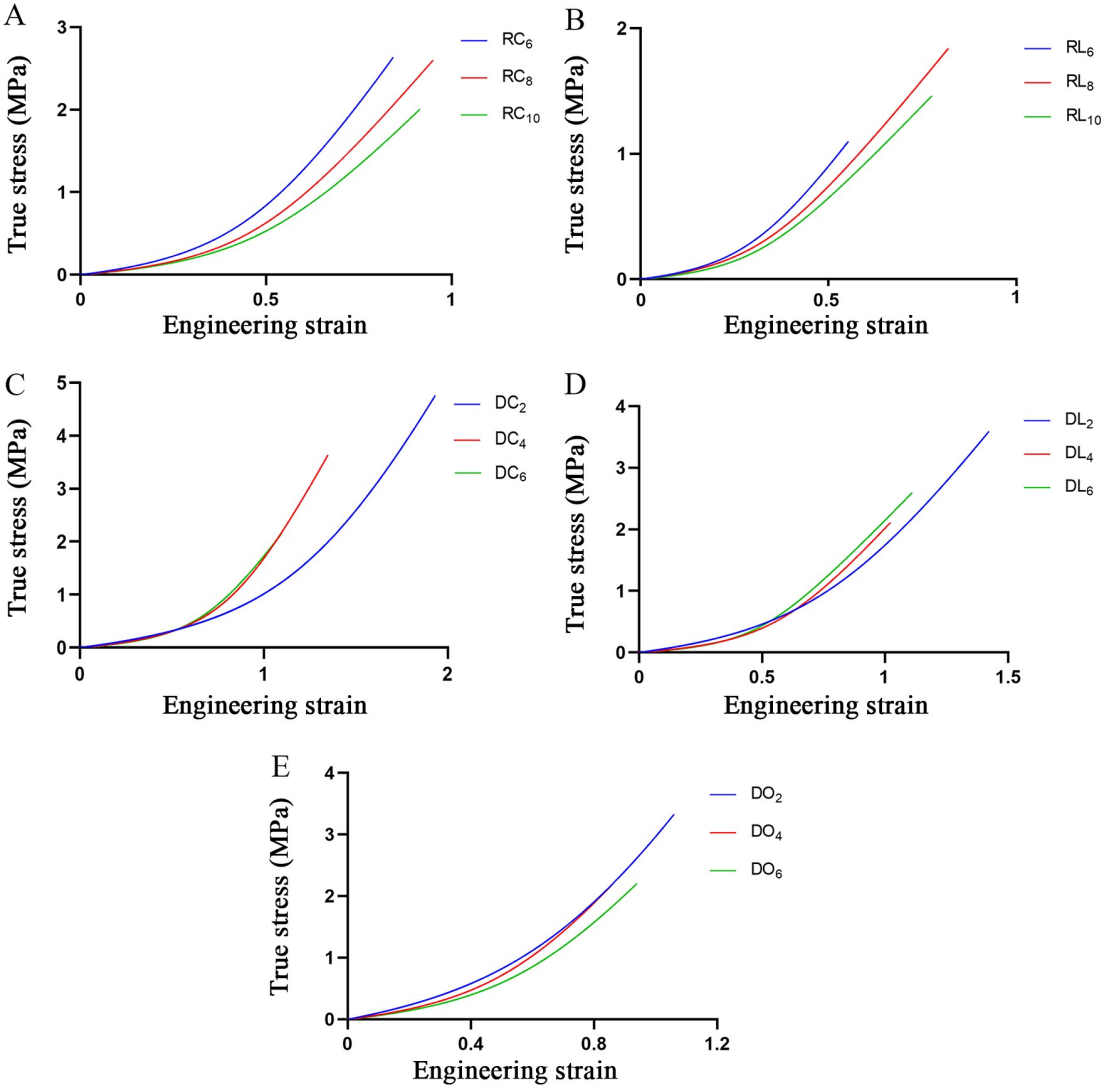
Fifteen model-generated stress-strain curves. (A) The groups of rectangular circumferential specimens. (B) The groups of rectangular axial specimens. (C) The groups of dog-bone-shaped circumferential specimens. (D) The groups of dog-bone-shaped axial specimens. (E) The groups of dog-bone-shaped oblique specimens.

The stress-strain curves for all the specimens indicated a satisfactory fit with the mathematical model, and the means of the parameters in the different groups are shown in Table 3. Fifteen model-generated stress-strain curves for each group, which were based on the mean values of the model parameters in Table 3, are shown in Fig 6.

**Table 3:** Mean model parameters in the different groups.

Group	K (mm^2^/N)	A	B (MPa)
RC_6_	0.142±0.041	0.525±0.074	0.318±0.072^a^
RC_8_	0.179±0.047	0.528±0.071	0.228±0.056
RC_10_	0.206±0.096	0.558±0.140	0.227±0.060
RL_6_	0.231±0.094	0.339±0.156	0.144±0.059
RL_8_	0.260±0.148	0.367±0.122	0.145±0.051
RL_10_	0.307±0.188	0.348±0.172	0.101±0.091
DC_2_	0.146±0.030	1.412±0.133^b^	0.666±0.088^b^
DC_4_	0.146±0.032	0.885±0.124	0.290±0.061
DC_6_	0.194±0.047	0.748±0.077	0.213±0.040
DL_2_	0.178±0.047	0.896±0.224 ^c^	0.524±0.273^c^
DL_4_	0.213±0.065	0.625±0.102	0.198±0.068
DL_6_	0.234±0.058	0.530±0.030	0.144±0.019
DO_2_	0.119±0.078	0.843±0.100 ^d^	0.897±0.407^d^
DO_4_	0.154±0.043	0.626±0.112	0.430±0.123
DO_6_	0.170±0.001	0.668±0.073	0.399±0.165

Note: Values are presented as the mean±standard deviation.

^a^ = p<0.05 against RC_8_ and RC_10_.

^b^ = p<0.001 against DC_4_ and DC_6_.

^c^ = p<0.01 against DL_4_ and DL_6_.

^d^ = p<0.05 against DO_4_ and DO_6_.

For the rectangular circumferential specimens, the difference was in parameter B. The B value of RC_6_ was significantly higher than the B values of RC_8_ and RC_10_. However, for the rectangular axial specimens, none of the parameters showed significant difference. For the dog-bone-shaped circumferential, axial and oblique specimens, the differences were in parameters A and B. The values of these parameters in the groups with 2-mm-wide specimens were significantly higher than the values in the other groups (Table 3).

The physical properties of each group, including the elastin and collagen fiber moduli (E_E_ and E_C_), ultimate strain (ε_u_) and ultimate stress (σ_u_), are shown in Table 4.

**Table 4:** Mean physical properties in the different groups.

Group	E_E_ (MPa)	E_C_ (MPa)	ε_u_	σ_u_ (MPa)
RC_6_	0.564±0.147^a^	6.938±2.003	0.841±0.114	2.633±0.592
RC_8_	0.407±0.119	5.503±1.436	0.939±0.086	2.598±0.396
RC_10_	0.381±0.096	5.512±2.781	0.890±0.120	1.998±0.539
RL_6_	0.485±0.285	4.482±2.021	0.569±0.226 ^h^	1.100±0.208 ^h^
RL_8_	0.404±0.203	4.294±2.097	0.754±0.187	1.838±1.009
RL_10_	0.258±0.117	4.249±2.771	0.676±0.082	1.462±0.938
DC_2_	0.442±0.043^b^	6.649±1.342	1.915±0.095^c^	4.702±0.377 ^c^
DC_4_	0.311±0.040	6.788±1.241	1.319±0.066 ^c^	3.638±0.578 ^c^
DC_6_	0.271±0.052	5.123±1.167	1.082±0.061 ^c^	2.142±0.395 ^c^
DL_2_	0.526±0.192 ^d^	5.433±1.672	1.370±0.453	3.594±1.766
DL_4_	0.289±0.059	4.751±1.399	0.948±0484	2.093±0.935
DL_6_	0.256±0.037	4.232±1.026	1.117±0.180	2.593±0.617
DO_2_	0.939±0.366 ^e^	10.309±4.827	1.049±0.180^f^	3.321±0.187 ^g^
DO_4_	0.618±0.114	6.278±1.688	0.818±0.181	2.149±0.961
DO_6_	0.539±0.194	5.355±0.225	0.935±0.112	2.207±0.226

Note: Values are presented as the mean±standard deviation.

^a^ = p<0.05 against RC_8_ and RC_10_.

^b^ = p<0.001 against DC_4_ and DC_6_.

^c^ = p<0.01 against the corresponding other two groups.

^d^ = p<0.01 against DL_4_ and DL_6_.

^e^ = p<0.05 against DO_4_ and DO_6_.

^f^ = p<0.05 against DO_4_.

^g^ = p<0.01 against DO_4_ and DO_6_.

^h^ = p<0.01 against DL_6_.

**Fig 7:**
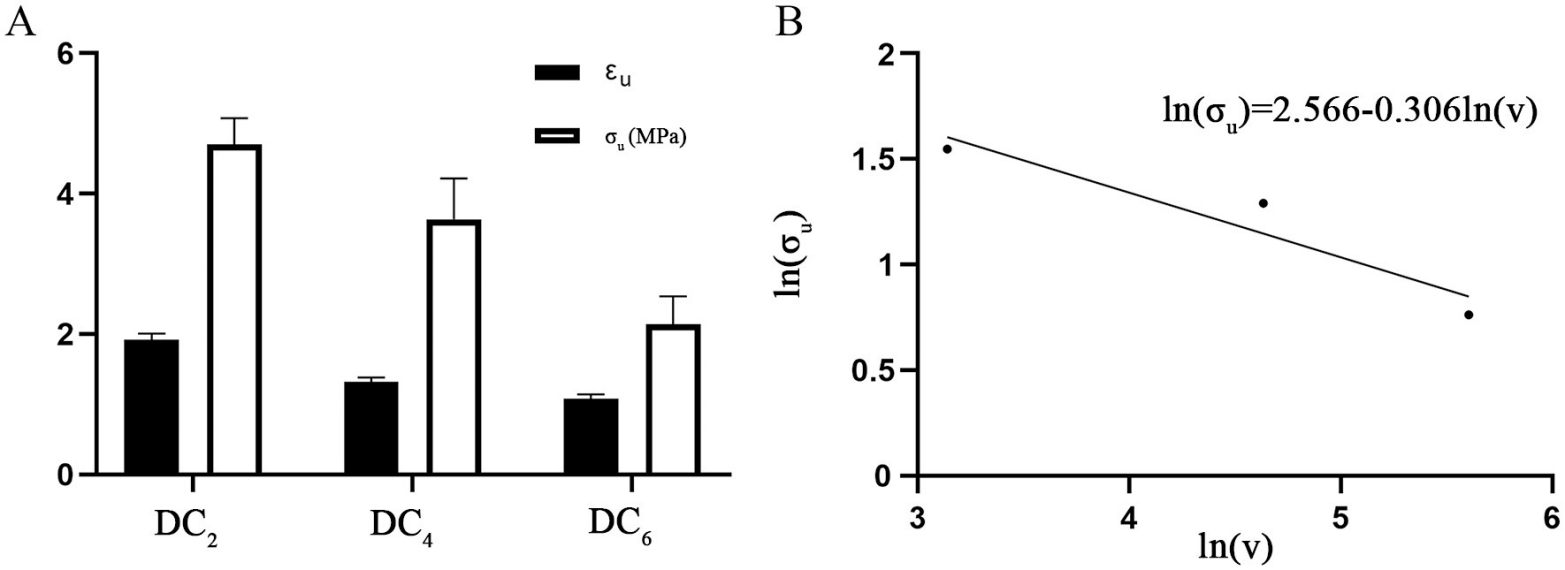
The ultimate values in the DC groups. (A) Comparison of ε_u_ and σ_u_ among the DC groups. (B) Logarithmic plot of the strength size effect of the DC groups.

For the rectangular circumferential specimens, the difference was in E_E_. The E_E_ value of RC_6_ was significantly higher than the values of RC_8_ and RC_10_. None of the four values showed significant difference for the rectangular axial specimens. For the dog-bone-shaped circumferential samples, the values of E_E_, ε_u_ and σ_u_ were different. The E_E_ value of DC_2_ was significantly higher than the values of DC_4_ and DC_6_. Moreover, the values of ε_u_ and σ_u_ were significantly different among the three groups: the values of DC_2_ were larger than those of DC_4_, and the values of DC_4_ was larger than those of DC_6_ (Fig 7A). For the dog-bone-shaped axial specimens, the difference was in E_E_, and DL_2_ showed a significantly higher E_E_ than DL_4_ and DL_6_. For the dog-bone-shaped oblique specimens, E_E_, ε_u_ and σ_u_ were different. The E_E_ and σ_u_ of DO_2_ were significantly higher than those of DO_4_ and DO_6_, whereas the value of ε_u_ of DO_2_ was significantly higher than that of DO_4_ (Table 4).

For RC_6_ and DC_6_, the ε_u_ of RC_6_ was lower than that of DC_6_, whereas the difference in σ_u_ was not statistically significant. For RL_6_ and DL_6_, the values of ε_u_ and σ_u_ of RL_6_ were 1.96 times and 2.36 times lower than those of DL_6_, respectively (Table 4).

The results from different directions were not compared because they were punched from different locations.

## Discussion

Although many aortic uniaxial tensile tests have been reported [14–32], no attention has been paid to the influence of the geometry and size of the specimens on the test results; hence, there are no corresponding experimental protocols. In this study, we systematically investigated the influence of the shape and size of the specimens on the mechanical properties of porcine aortas by using custom-designed tissue cutters, clamps and molds and explored the optimal experimental conditions and parameters of material tests.

### The use of custom-designed tissue cutters

In some published studies [37, 42, 43] with figures of aortic uniaxial tensile test specimens, the edges of the specimens are not sufficiently smooth or have notches, which will produce stress concentrations in these zones. The magnitude of these stress concentrations is usually expressed in terms of the stress-concentration factor K. K is independent of the material properties; rather, it depends only on the geometry and the type of discontinuity. The stress concentrations caused by sharp notches in specimens can lead to fracture at lower nominal stresses [44]. In our study, custom-designed cutters could ensure smooth specimen edges and avoid potential stress concentrations.

### The use of custom-designed clamps and molds

Another important factor for uniaxial tensile tests of soft tissue is the rapid clamping of the specimens and ensuring that the samples will not slip and fail in the clamping place. In some studies [14, 15, 21, 22, 24, 29], the samples were clamped on grips using sandpaper. In other studies [26, 27, 31], the specimens were sandwiched between the clamps using sandpaper and glue. Our preliminary experiments showed that with the clamping method mentioned above, the strips easily slipped off when the clamping pressure was low or broke from the clamping point when the pressure was high. In the present study, custom-designed clamps were used to fix the strips. The gap between the clamps was 1.5 mm (the aorta was approximately 2 mm in thickness) (Fig 2C and 2D). The mechanical clamping of the clamps and the chemical adhesion of the glue ensured that the specimens did not slip and were not damaged by clamping; moreover, this process ensured that the samples did not fail at the clamps. Custom-designed molds with scales were utilized to fix the aspect ratio of the test zone and to quickly finish the preprocessing (Fig 2E and 2F).

### The influence of the shapes of specimens

In previous studies, the shape of the strips was either rectangular or dog-bone-shaped, with a width ranging from 2 mm to 10 mm (Table 1). This is the reason why the shape and size of the specimens were chosen in this study. Rectangular specimens were not obliquely stamped because the lower diameter of the distal aorta made it difficult to ensure the corresponding aspect ratio.

Sang Chao, et al. [45] studied the relationship between the specimen geometry and clamping conditions and the failure point, and their results showed that mid-sample failure occurred in 94% of dog-bone specimens, whereas this type of failure occurred in only 14% of the rectangular samples, which is similar to our findings. However, the effects on the mechanical parameters were not clarified in their study. According to our research, 88.9% of rectangular specimens failed close to the clamps, and the ultimate strain and stress of DL_6_ were 1.96 and 2.35 times those of RL_6_, which had the same width and aspect ratio but different shapes. The reason for this phenomenon was mainly attributed to Saint-Venant’s principle, which essentially states that the stress and strain produced at points in a body sufficiently removed from the region of load application will be the same as the stress and strain produced by any applied loads that have the same statically equivalent resultant and are applied to the body within the same region [46]. The principle shows that in the uniaxial tensile test, the stress in the zone far away from the clamping positions is relatively uniform, and the stress near the clamping positions is uneven, so the specimens are prone to fail at the uneven stress location when the load is low. For the dog-bone-shaped specimens, the cross-sectional area near the clamping positions is relatively large, so the stress near the clamping positions with uneven stress is smaller than that in the test zone, making the stress in the test zone uniform and the failure point occur there. Because rectangular strips are easier to take and require lower aortic dimensions compared to dog-bone-shaped strips, rectangular strips are by fare the best option if video-extensometer is used in the central area with uniform stress for aortic uniaxial tensile test that measure physiological range and do not require stretching to rupture [38, 47]. However, when it comes to tension to rupture, rectangular samples are unsuitable for uniaxial tensile tests.

### The influence of the sizes of specimens

Until now, there have been no comparative studies on the size effects of aortic walls or other biological specimens. This study shows that the sizes of the specimens have apparent influences on the aortic mechanical properties, regardless if the specimens are rectangular or dog-bone-shaped. This effect is most obvious in the dog-bone-shaped circumferential specimens, and the ultimate strain and stress decrease with the increase in specimen width among the three groups (Fig 7A). The results should be attributed to the microstructure of the aortic wall. The aorta is composed of the intima, media and adventitia. The intima consists of a single layer of endothelial cells, and it is mechanically negligible in healthy young individuals. The media is the middle layer of the artery and consists of a complex three-dimensional network of smooth muscle cells and elastin and collagen fibrils [48]. M.K. O’Connell et al. [49] applied electron and confocal microscopy techniques to obtain 3D volumetric information of aortic medial microstructures and found that the media consisted of several concentric lamellar units bound together, each containing smooth muscle cells, whose long axis was radially tilted and angled more closely to the circumference and surrounded by collagen fibers embedded in the extracellular matrix. The adventitia consists mainly of fibroblasts and fibrocytes, histological ground substance and thick bundles of collagen fibrils forming fibrous tissue. The media and adventitia are composed of an isotropic matrix, and two families of collagen fibers helically wound along the arterial axis and symmetrically disposed with respect to the axis. The anisotropy in the mechanical response is induced by collagen fibers, so their response is orthotropic and can be considered a fiber-reinforced material [34, 48]. Collagen fibers of the two families are nearly symmetrically arranged with respect to the cylinder axis and are closer to the circumferential direction in the media but closer to the axial direction in the adventitia [50]. Moreover, the elastic properties of the media and adventitia are different, and the media is much stiffer than adventitia [51–53]. Therefore, for the intact aortic wall, the media should be the main passive load bearing, in which collagen fibers are close to the circumferential direction. Hence, the circumferential stretch of the artery wall is equivalent to the axial stretch of the fiber-reinforced material. For the fiber-reinforced material, the longitudinal strength is size dependent, and the strength of the material decreases with increasing diameter; however, the longitudinal modulus does not change appreciably [54]. For brittle materials, it is generally believed that the probability of failure increases as the specimen size increases under the same stress conditions [55, 56]. The strength size effects of fiber-reinforced plastic (FRP) composite materials and structures have also been reported in other studies [57–60]. The weakest-link theory was first proposed by Pierce [61], and Weibull [62] made great progress in this subject. This theory is usually used to analyze the strength size effects. The theory assumes the material is made up of smaller elements linked together and that failure of the material as a whole occurs when any of these elements or links fail. If the strength distribution of specimens is in accordance with Weibull theory, the strength values may be related to sample size. This can be expressed as follows:

$$\frac{\sigma_{2}}{\sigma_{1}}={\left(\frac{V_{1}}{V_{2}}\right)}^{\frac{1}{m}}$$

where σ and V are the strength and volume of the specimens, respectively, and m is the shape parameter. The equation expresses the relationship between stress and volume and thus quantifies the size effect. A logarithmic plot of stress versus volume gives a straight line relationship of slope -1/m. A detailed derivation is given in reference [59]. Fig 7B shows a logarithmic plot of stress versus volume of the DC groups with the fitting equation ln(σ_u_) = 2.566 − 0.306 ln(V). As shown in Fig 7B, the ultimate stress in the DC groups is basically consistent with Weibull theory. Hence, the size effect of the aorta can be explained by the Weibull theory.

### The influence of the shapes and sizes of specimens

It is speculated that the rectangular circumferential specimens failed under a smaller load due to Saint-Venant’s principle, so they do not exhibit the same size effect as the dog-bone-shaped specimens. The ultimate stress of DC_6_ was less than that of RC_6_, but there was no statistically significant difference between them. This could be explained by Weibull theory and Saint-Venant’s principle. According to Weibull theory, the ultimate stress of RC_6_ (aspect ratio 3:1) would be higher than that of DC_6_ (aspect ratio 4:1). Moreover, rectangular specimens will easily fail at the uneven stress location near the clamping position under lower load due to Saint-Venant’s principle. As a result, the RC_6_ group with a small volume ruptured in advance. Therefore, the specimens should be processed into dog-bone shapes, and the aspect ratio of the test area must be reasonable and consistent for uniaxial tensile tests of aortas.

### The optimal specimen choice for aortic uniaxial tensile testing performed until rupture

The E_E_ values of the 2-mm-wide dog-bone-shaped groups and the 6-mm-wide rectangular circumferential groups were higher than those of the corresponding other groups, whereas the values of E_C_ did not show a significant difference. This may be because the width of the test zone of specimens was relatively small, making them more sensitive to load errors at the beginning of the uniaxial tensile test. The parameters (A and B) of the 2-mm-wide groups were different from those of the 4-mm-wide and 6-mm-wide groups, and the 6-mm-wide dog-bone-shaped groups required a larger tissue size. Hence, the 4-mm-wide dog-bone-shaped group was more suitable for the aortic uniaxial tensile tests performed until rupture.

### Limitations and future directions

The layer specificity of the aorta was not explored in our study for several reasons. (1) The aorta functions as a whole under the physiological states, and the mechanical properties of the whole layer are not a simple addition of three layers. (2) In the field of forensic injury biomechanics, layer-specific research on mechanical properties has little significance since these injuries are usually manifested as whole-layer rupture or dissection or other dangerous events. (3) This study is mainly focused on the effects of geometry and size on porcine aortic material properties and how to generate ideal material properties through our custom-designed instruments. In future research, we will use the optimized experimental method to test human aortas in a systematic group and explore how to process specimens and obtain accurate mechanical parameters for aortic tissues with relatively small lesions.

## Conclusions

The study showed that sample geometry and size affect aortic uniaxial tensile tests. The rectangular specimen was not suitable for aortic uniaxial tensile testing performed until rupture according to Saint-Venant’s principle. The size effect of the aorta conformed to Weibull theory. According to the results, dog-bone-shaped specimens with a width of 4 mm were the optimal choice for aortic uniaxial tensile testing performed until rupture.

## Supporting information

S1 Dataset Raw data of uniaxial tension test. (ZIP)
